# The Predictors of Negative and Positive Affect among People with Dementia: A Cross-Sectional Study

**DOI:** 10.3390/medicina59101724

**Published:** 2023-09-27

**Authors:** Mohammad Rababa, Ayham Aldrawsheh, Audai A. Hayajneh, Anwar M. Eyadat, Raghad Tawalbeh

**Affiliations:** 1 Adult Health Nursing Department, Faculty of Nursing, Jordan University of Science and Technology, P.O. Box 3030, Irbid 22110, Jordan; aahayajneh@just.edu.jo (A.A.H.);; 2 Community and Mental Health Department, Faculty of Nursing, Jordan University of Science and Technology, P.O. Box 3030, Irbid 22110, Jordanameyadat@uwm.edu (A.M.E.)

**Keywords:** negative and positive affect, emotional closeness, agitation, dementia, Jordan

## Abstract

*Background and Objectives*: This cross-sectional study examined the predictors of negative and positive affect among individuals with dementia. *Materials and Methods*: A sample of 102 Jordanian participants diagnosed with dementia was recruited from residential care facilities, and data were collected using different measures. *Results*: The results revealed that higher levels of negative affect were significantly associated with increased physical and verbal agitation among individuals with dementia. Conversely, lower levels of positive affect were associated with residing in a nursing home. *Conclusions*: These findings highlight the importance of recognizing the impact of both negative and positive affect on the well-being of individuals with dementia. Interventions targeting the reduction of negative affect and promoting positive affect could alleviate agitation and enhance emotional closeness in this population.

## 1. Introduction

Dementia is considered a gained cognition failure in multiple domains adequately extreme to impact psychological or social well-being and is typically paired with more than one neuropathology [1]. Dementia is related to various illnesses and injuries that affect the brain as a primary or secondary target. It causes a degeneration in cognitive abilities beyond the typical consequences of natural aging, making older people the main affected population [2]. At least 55 million individuals have dementia globally, with about 10 million new patients yearly. Among all diseases, dementia is the seventh most common cause of death and one of the important causes of disability and dependence among older adults worldwide, leading to many physical, psychological, economic, and social consequences for people with dementia and their caregivers, relatives, and community as whole [2]. The prevalence of dementia is more significant in females than males and in individuals aged 60 to 69 than in older ones [3].

Dementia is characterized by cognitive impairment and noncognitive symptoms, known these days as neuropsychiatric symptoms (NPSs). Aggression and agitation are examples of NPSs correlated with cognitive decline, poor outcomes, and loss of independence. These are a source of danger for patients and caregivers since they may hurt themselves, their caretakers, or other patients [4,5]. A cross-sectional study showed that about 90% of the nursing home (NH) residents with dementia suffer from at least one NPS, 88% demonstrate marked agitation, and 56% express dementia-related apathy, with no gender variations seen. Apathy, non-physically aggressive behavior, and physically aggressive behavior were more prevalent in patients with extreme cognitive deterioration; the latter was associated with low levels of awareness and aberrant motor behavior, while verbally agitated behavior was expected in patients in mild and moderate stages of dementia. Negative affect is one of the neuropsychiatric symptoms related to irritability and agitation/aggression experienced by people with dementia (PWDs) [6].

Caring for PWDs is still associated with many challenges rather than aggressive behaviors. Caregivers still need to know how to care for challenging PWDs appropriately. Experiencing fewer bad days may make the caregiving role more manageable, with less fluctuation in the caregiver’s negative affect. Fewer daily stressors, more positive events, higher than average daily care-related stressors or duration of caregiving, not being a spouse, and less than an average dependency of individuals with dementia with regard to activities of daily living were associated with less intrinsic fluctuation [7].

Social interactions are critical to human well-being, especially when they lead to positive affect. Evidence shows that the nature of social interactions in the context of close social partners contributes to daily experiences of positive and negative affect and the levels of relationship satisfaction [8]. Additionally, social ties and connectedness positively enhance physical and mental health, being associated with improved physical activity, less sedentary lifestyle, and better mood [9]. Older people with diverse ties were likely to behave differently [9]. At NHs, PWDs are at higher risk of engaging in less social interaction and having poorer well-being [10]. If given a choice, they would prefer to live in their homes instead of nursing homes and conduct meaningful interactions with narrow, close social networks instead of large ones because of a higher sense of privacy and security. Al Ghassani and Rababa [11] found no significant associations between core network size or closeness and PWDs’ activities of daily living (ADLs) and instrumental activities of daily living (IADLs), the number of ADLs and IADLs provided by the caregiver, and affect and agitation among PWDs [12]. However, this study recruited a small sample size and included not normally distributed data. Since PWDs face several challenges to achieving positive social relations, they are associated with more heightened interest in and satisfaction within and between NH residents with dementia. Such relations predict negative affect and sadness among PWDs [13]. Both verbal and nonverbal interactions significantly correlate with positive and negative emotional expressions. Notably, positive interactions are associated with more positive emotional expressions, emphasizing the importance of social interactions for PWDs to promote their psychological well-being [10].

At NHs, there are some gender differences; males and females are treated differently or at least perceive NH issues differently. Males complain significantly less than female residents [14]. A previous study showed gender differences in behavioral and psychological symptoms of dementia. For example, males had a higher prevalence of apathy and sexually inappropriate behavior than females, while females had a higher prevalence of anxiety and sadness than males [15]. Another study showed that there was a difference based on gender regarding depressive symptoms. Females exhibited depressive symptoms such as anxiety, sadness, and somatic complaints more frequently than males. By contrast, males exhibited more aggressive behavior and were more likely to get anticonvulsants than females [15].

Given the continuing increment in dementia cases and different emotional and social closeness statuses between NH and community residents, little attention has been given to the association between emotional closeness and agitation among PWDs. There is a need to assess the associations of negative and positive affect with emotional closeness and agitation among PWDs. This examination will provide nurses and other care providers with solid baseline knowledge for assessing persons’ emotional problems, understanding how to provide appropriate care, and designing effective interventions to enhance PWDs’ quality of life. Accordingly, this study aims to examine the association of emotional closeness and agitation with positive and negative affectivity in older adults with dementia residing in the Jordanian community or NHs.

## 2. Methods

### 2.1. Study Design, Setting, and Sample

This descriptive correlation cross-sectional study was conducted on a convenience sample of 102 Jordanian PWDs. Three nursing homes and healthcare centers located in northern Jordan were selected. The sample size was specified using a G-power calculator, entering the following values: a significance level of 0.05, three predictors, a power level of 80%, and an anticipated effect size of 0.15. The sample size was adequate for a significant multiple regression to investigate the association of negative and positive affect with emotional closeness and agitation among PWDs. The qualified participating PWDs in the current study were (1) 65 years and older [16] (2) were diagnosed with dementia based on the Montreal Cognitive Assessment (MoCA). PWDs taking antipsychotic medications were excluded from the study.

### 2.2. Measures

Dementia. Dementia was measured using the MoCA designed by Nasreddine et al. [17]. The MoCA was utilized to estimate the degree of dementia by assessing the eight parts of cognitive functions. The entire potential score of the MoCA varies from 0 to 30. The cutoff point for a diagnosis of dementia is a MoCA score of less than 26, with lower scores suggesting a greater degree of dementia. As recorded in their medical documents or documented by their caretakers, older adults with psychiatric issues were excluded from the study.

Agitation. The Cohen-Mansfield Agitation Inventory (CMAI), a 29-item scale designed by Cohen-Mansfield et al. [18], was utilized to evaluate the agitated behaviors of PWDs by their guardians according to the frequency with which they arise. In this study, these agitated behaviors are classified into physically aggressive, physically non-aggressive, verbally aggressive, and verbally non-aggressive since the CMAI does not have a total score. Therefore, each category was a dependent variable in the present study. Each category is ranked on a 7-point scale, varying from (1) never to (7) several times per hour. The average internal consistency reliability of the CMAI is κ = 0.92 (0.88–0.92) [18].

Affect. The Positive and Negative Affect Schedule (PANAS) is a 20-item self-report questionnaire developed by Watson et al. [19] consisting of various items that define participants’ positive and negative sensations, feelings, relations, connections, and the hardships they confront in life [20]. These items are ranked on a 5-point Likert scale varying from 1 (very slightly, not at all) to 5 (extremely). Watson et al. [19] documented Cronbach’s alpha scores for positive items, which varied from 0.86 to 0.90, and for negative items, which varied from 0.84 to 0.87.

The CMAI and PANAS were translated into Arabic and cross-checked with a university professor in English. The CMAI and PANAS were left with the guardians for a week. The guardians observed PWDs for positive and negative affect and agitated behaviors. The researcher instructed the guardians on administering these tools (CMAI and PANAS).

Emotional closeness. The Social Convoy Questionnaire (SCQ) created by Kahn and Antonucci [21] was employed to estimate emotional closeness by utilizing circle diagrams to determine the degree of closeness to a PWD based on the circle selected. The SCQ has three circles: inner, middle, and outer. The inner circle represents “Feel very close, so close that it would be hard to imagine life without”. The middle circle represents “Do not feel quite so close as those in the inner circle but are still very close”. The outer circle represents “Feel less close but who are still important”. The internal consistency and test–retest reliability of the SCQ were examined among a sample of community-dwelling older adults and found to be 0.71 and 0.80, respectively [22].

Demographic data. The participants’ demographic characteristics were gathered by self-report and validated by reviewing medical documents or guardians’ self-reports. These characteristics include age, gender, religion, marital status, educational level, monthly household income, employment status, and any family members with medical/health professional backgrounds.

### 2.3. Pilot Study

Before carrying out the present study, the researcher conducted a pilot study on 14 PWDs and their guardians to consider the feasibility of the Arabic versions of the CMAI and PANAS, to decide if any changes were required to the tools and to assess the clearness and understanding of the items from the patient’s viewpoint [23]. Those 14 participants were chosen conveniently from a large nursing home and healthcare center in Irbid, Jordan. Ethical approval from the research ethics committee of the nursing faculty was acquired before the pilot study. The results of this pilot study revealed that the Arabic version of the CMAI and PANAS has adequate internal consistency scores, varying from Cronbach’s α = 0.89 to α = 0.67. The “Alpha if item deleted” procedure was employed to investigate individual items to establish how each item influenced the scale’s reliability [24] and was utilized for item improvement.

Nevertheless, no item omissions enhanced Cronbach’s alpha of the tools. Thus, all study tool items were utilized in the present study. Third, concerning the clearness of the tools, some participants suggested that a few items were vague or hard to answer. Therefore, the researcher paraphrased some items and added clarifying words for others.

### 2.4. Ethical Considerations

The ethical approval (IRB# 657-2021) for this study was acquired from the University and the managers of the chosen healthcare centers and nursing homes. Written consent was received from the participants with mild dementia, while for those with moderate to severe dementia, their assent was acquired to participate in this study. The researchers asserted the confidentiality and privacy of the collected data throughout the study’s procedure.

### 2.5. Data Collection

The study data were gathered using medical documents and observational and self-report tools. The admission and registration office at each participating NH and healthcare center was reached to obtain a checklist of potentially eligible participants. The researcher met with the potentially eligible participants and acquired written consent from those lawfully and cognitively able to provide their consent. For those participants who could not verbally self-report or make their own decisions, their guardians provided written consent, and the participants provided verbal assent. All participants providing assent or consent had the opportunity to have their questions answered. After obtaining the finished questionnaires, each participant was given a digital code to protect the participant’s identity. Then, the researcher’s personal computer was used to save the collected data in sealed files.

### 2.6. Statistical Data Analysis

The statistical data analysis was performed employing the IBM SPSS V.21 program. The significance level was specified at 0.05 throughout the analysis. The means and standard deviations were employed to describe the continuous variables, and the frequency and percentages for the categorical variables. The Kolmogorov–Smirnov (KS) test of statistical normality was employed to evaluate the statistical normality of the study variables. Pearson’s (r) correlation test was used to determine the study variables’ association. Multiple regression analyses were employed to examine the association between the study variables. In the regression analysis, we used the best subsets regression procedure to find the best subset with a fixed number of variables for multiple linear regression with the minimum sum of squared error (SSE) and largest R2, as well as to rule out multicollinearity between independent and dependent variables.

## 3. Results

### 3.1. Correlations between Study Variables

Table 1 shows that controlling for age and gender, patients’ positive affect correlated significantly and positively with their non-aggressive physical behavior score, r = 0.260, *p* < 0.010. Controlling for age and gender, patients’ physical aggressiveness mean scores correlated significantly and positively with their mean negative affect scores, r = 0.556, *p* < 0.010, but not with their mean positive affect scores. Moreover, controlling for age and gender, patients’ verbal aggressiveness mean scores correlated significantly and positively with their negative affect score, r = 0.616, *p* < 0.010. However, positive affect scores did not correlate significantly with the patients’ verbal aggressiveness scores. Likewise, patients’ non-verbal aggressiveness scores correlated significantly and positively with their mean negative affect scores but not with their positive affect scores. Unexpectedly, patients’ positive and negative affect scores were not correlated significantly, with a *p*-value = 0.732.

#### 3.1.1. Predictors of Negative Affect

In a multivariate linear regression analysis of the PWDs’ mean negative affect scores (Table 2), the type of setting where the PWDs live did not correlate significantly with their mean negative affect scores. Also, the patients’ ages (in years) did not correlate significantly with their negative affect scores. However, male PWDs had significantly higher mean negative affect scores than females, with a beta coefficient = 3.785 and *p*-value = 0.010. The multivariate analysis showed that the widowed or divorced PWDs had significantly higher mean negative affect scores than the married or single PWDs, with a beta coefficient = 3.081 and *p*-value = 0.038. However, PWDs’ physically aggressive behavior correlated significantly and positively with their negative affect mean scores. Physically aggressive dementia patients measured significantly greater mean negative affect scores than those not identified to exhibit physical, aggressive behaviors, with a beta coefficient = 6.302 and *p*-value < 0.001. Nevertheless, according to the multivariate analysis, the PWDs’ physical non-aggressiveness scores, Montreal cognitive function scores, and social convoy scores did not correlate significantly with their mean negative affect scores. Still, PWDs with verbal agitation measured significantly greater negative affect scores than those with no verbally aggressive behaviors according to the CMAI questionnaire classification, with a beta coefficient = 2.662 and *p*-value = 0.019.

#### 3.1.2. Predictors of Positive Affect

As seen in Table 3, the multivariate linear regression analysis showed that PWDs residing in NHs had significantly lower mean positive affect scores than those living in the community (*p*-value = 0.008). Also, male PWDs measured significantly higher mean positive affect scores than females (*p*-value = 0.017). Moreover, PWDs with higher monthly income measured significantly lower, which indicates a positive affect score (*p*-value = 0.001). Furthermore, PWDs with higher educational levels measured significantly higher mean positive affect scores than those with a lower educational level (*p*-value = 0.033). Moreover, PWDs with a family member in the healthcare field measured significantly greater mean positive affect scores (*p*-value = 0.028).

## 4. Discussion

The current study showed that social networks and emotional closeness did not correlate significantly with measured agitation, affect, or cognitive function scores. These results are consistent with other studies which did not show a significant correlation between the closeness and size of core networks and density of care in PWDs and instrumental activities of daily living (ADLs) and non-instrumental activities of daily living (IADLs) [11,12]. However, The current results are inconsistent with other studies [25,26] that showed social interactions being significantly correlated with enhanced cognitive function, alleviating agitation symptoms. These results could be related to the correlation between emotions and cognitive function, indicating that older adults need emotional support from family, relatives, and friends to prevent cognitive function deterioration.

This study showed that positive affect did not correlate significantly with PWDs’ age, physically aggressive, physically non-aggressive, verbally aggressive behaviors, and cognitive function (MoCA). These results are not consistent with previous studies [27,28,29], which showed that positive affect scores correlated significantly with the likelihood of not developing agitation and decreasing cognitive decline.

### 4.1. Predictors of Positive Affect

This study showed that the patient’s sex was correlated significantly with their mean positive affect score; male patients had a significantly higher mean positive affect score than females (*p*-value = 0.017). This result is consistent with another study [28], which found that married men with higher positive affect scores are more likely to be cognitively and socially active. Therefore, this result is inconsistent with another study that found females scoring significantly higher than males in depression and overall level of positive affect [30]. This finding may be explained by the fact that females commonly use more inadequate coping strategies (such as escape from problem-solving and denial) than males [31].

In the present study, we found that those with higher educational levels had significantly higher mean positive affect scores compared to the patients with lower academic levels on average (*p*-value = 0.033). The result is consistent with other studies that have found that people with higher educational levels correlated slightly positively with a lower incidence of dementia [28]. Our findings may be explained by the fact that educational level protects against cognitive function impairment [29,32]. In my opinion, the reason is mainly due to the stressors and depression related to the conditions of life and poverty, which contributes to the low education level and abstaining from seeking knowledge; all of these reasons lead to the deterioration of health over time. This study found that PWDs with a family member in the healthcare field had significantly greater mean positive affect scores than those with no healthcare/medical family members (*p*-value = 0.028). The result is consistent with other studies that indicate a positive correlation between having a caregiver working in the healthcare field close to dementia patients and partial relief of symptoms of agitation [33]. The result is that the presence of caregivers with a health professional background helps strengthen social interactions, which improves psychological and mental health. In addition, their assistance in fulfilling unmet needs protects PWDs against psychological and physical harm.

### 4.2. Predictors of Negative Affect

The findings of this study suggest that several factors may contribute to the negative affect scores of patients with dementia (PWDs). The results showed that the type of setting where PWDs live, and their age did not significantly correlate with their mean negative affect scores. However, this study and earlier studies support that male PWDs had significantly higher mean negative affect scores than females, while emotional regulation is different based on gender [31]. For example, males are more likely to use reappraisal to improve their mood compared to females [31]. Moreover, widowed or divorced PWDs had significantly higher mean negative affect scores than married or single PWDs [7,34,35,36]. Thus, this result highlights the need for healthcare providers and caregivers to consider the patient’s marital status and gender when assessing and managing their negative affect.

Furthermore, the results show that PWDs who exhibit physical aggressiveness have significantly higher mean negative affect scores than those who do not, as supported by previous studies [37,38,39,40]. This finding suggests that physical aggression may be an essential factor contributing to the negative affect of PWDs [38,40]. In addition, an early study by Hwang et al. [38] suggested that physical aggression is a neuropsychiatric symptom common in PWDs with mild cognitive impairment. The study found that PWDs with mild cognitive impairment and neuropsychiatric symptoms had significantly higher negative affect levels than those without. In addition, the authors suggest that physical aggression may contribute to the negative affect experienced by PWDs with mild cognitive impairment [38].

In addition, a recent systematic review suggests that physical aggression is associated with negative affect in PWDs and can have detrimental consequences for the patient and their caregivers [3]. Therefore, healthcare providers and caregivers should consider preventing and managing physical aggression as a critical aspect of care for PWDs. Healthcare providers and caregivers should consider this when managing aggressive behaviors in PWDs and aim to reduce or prevent physical aggression in these patients, which could be done using non-pharmacological interventions, including behavioral therapy, environmental modifications, and caregiver training [3].

Interestingly, the current study found that PWDs’ physical non-aggressiveness scores, Montreal cognitive function scores, and social convoy scores did not significantly correlate with their mean negative affect scores. A recent study concluded that physical aggression significantly predicted behavioral and psychological symptoms in dementia (BPSD), including negative affect, in PWDs. However, the review did not find significant associations between non-aggressive behavior, cognitive function, or social support and BPSD [41]. This result suggests that these factors may not be as important in predicting the negative affect of PWDs as other factors, such as physical aggression or marital status. Further, this highlights the need for tailored interventions for individual patients, which includes non-pharmacological interventions such as behavioral therapy, environmental modifications, and caregiver education [40].

Finally, the current study found that PWDs with verbal agitation had significantly higher negative affect scores than those without verbally aggressive behaviors. This result suggests that verbal aggression may also contribute to negative affect in PWDs. Healthcare providers and caregivers should take steps to reduce or prevent verbal aggression and improve the quality of life of people with dementia by developing effective management strategies, such as identifying and addressing the underlying causes of aggressive behavior, unmet needs, pain, and environmental factors. Moreover, the findings of this study support the idea that verbal aggression can contribute to negative affect in people with dementia and emphasize the importance of preventing or reducing verbal aggression in dementia care, in line with previous studies [42].

In summary, the findings of this study have important implications for healthcare providers and caregivers working with PWDs. The results suggest that gender, marital status, physical aggression, and verbal agitation should be considered when assessing and managing negative affect in PWDs. Furthermore, the findings highlight the need for further research to better understand the complex relationships between these factors and negative and positive affect in PWDs.

## 5. Limitations

This study is the first descriptive correlational study conducted in Jordan to examine the association of emotional closeness and agitation with positive and negative affectivity in older adults with dementia. However, this study has several limitations, including the use of a relatively small sample size and a convenience sample, which may threaten the internal validity and generalizability of the findings. Also, this study might not control for some unexpected confounding variables, such as pain and unexpected visitors, thus limiting the generalization of the study findings. Furthermore, this cross-sectional study does not establish a causal relationship between the dependent and independent variables since it is a descriptive correlational study. Moreover, the study was based on self-report measures, which may be subject to recall bias or social desirability.

## 6. Implications

The study of the association of negative and positive affect with emotional closeness and agitation among people with dementia has several implications for research, clinical practice, education, and policy. These implications include:

### 6.1. Research Implications

Further research is needed to explore the underlying mechanisms and causal relationships between emotional closeness, agitation, and negative and positive affect in PWDs and to identify effective interventions and strategies to manage agitation and promote positive affect in PWDs. Also, longitudinal studies can better understand how these factors evolve and their impact on the progression of dementia and overall well-being. Moreover, studies should examine the influence of cultural factors on emotional closeness, affect, and agitation in dementia, as cultural differences may play a significant role.

### 6.2. Clinical Implications

In terms of clinical implications, healthcare professionals should consider emotional closeness and agitation as critical factors when assessing and managing the emotional well-being of PWDs. In addition, caregivers and healthcare professionals should receive training on effective communication techniques and strategies to enhance emotional closeness and positive affect in PWDs. In addition, non-pharmacological interventions, such as behavioral therapy, environmental modifications, and caregiver education, should be implemented to reduce agitation and promote positive affect in PWDs.

### 6.3. Educational Implications

With regard to educational implications, educational programs for healthcare professionals and caregivers should include training on understanding and managing emotional closeness, agitation, and affective symptoms in PWDs. Additionally, dementia education should emphasize the importance of creating supportive and emotionally nurturing environments for PWDs. Finally, students pursuing healthcare professions should receive comprehensive training on dementia care, including knowledge of emotional well-being, affect, and agitation management.

### 6.4. Policy Implications

In terms of policy implications, policymakers should prioritize funding for research on dementia care, including studies investigating the impact of emotional closeness, agitation, and affective symptoms on the well-being of individuals with dementia. Furthermore, policies should support developing and implementing evidence-based interventions and training programs that address emotional well-being, affect, and agitation in dementia care.

In summary, the study’s findings have important implications for advancing research, improving clinical practice, enhancing educational programs, and informing policy development in dementia care. By addressing emotional closeness, agitation, and affective symptoms, healthcare providers and caregivers can better support the well-being and quality of life of PWDs.

## 7. Conclusions

This study explored the predictors of negative and positive affect in PWDs. Multivariate linear regression analysis revealed several significant findings. Regarding negative affect, the type of setting and age did not significantly correlate with mean negative affect scores. However, male patients had significantly higher scores than females, and widowed or divorced patients scored higher than married or single PWDs. Physical aggression and verbal agitation were also significant predictors of negative affect. On the other hand, positive affect scores were significantly lower for PWDs residing in NHs compared to those living in the community. Male PWDs and those with higher educational levels had significantly higher positive affect scores. PWDs with higher monthly income also had lower positive affect scores. These findings have important implications for healthcare providers and caregivers collaborating with PWDs. This discussion ultimately contributes to understanding the complex relationships between several factors and their impact on the emotional well-being of PWDs. By considering gender, marital status, physical aggression, and verbal agitation, healthcare providers and caregivers can better assess and manage negative affect in PWDs, improving their quality of life. Further research is warranted to investigate these factors and their implications for dementia care.

## Figures and Tables

**Table 1:** Bivariate Pearson’s correlations between measured concepts/outcomes.

	NPA	PA	VA	NVA	SCQ	MoCA	PosA	NegA
Physically non-aggressive (PNA)	1							
Physically aggressive (PA)	0.583 **	1						
Verbally aggressive (VA)	0.381 **	0.624 **	1					
Verbally non-aggressive (VNA)	0.755 **	0.619 **	0.449 **	1				
Emotional closeness (SQC convoy)	−0.008	−0.181	−0.050	−0.096	1			
Cognitive assessment (MoCA)	−0.130	−0.053	−0.051	−0.094	−0.150	1		
Positive affect subscale (PosA)	0.037	0.007	0.157	0.029	0.042	0.099	1	
Negative affect subscale (NegA)	0.245 **	0.552 **	0.610 **	0.376 **	0.024	−0.023	−0.070	1

** Correlation is significant at the 0.01 level (2-tailed).

**Table 2:** Multivariate linear regression analysis of the dementia patients’ negative affect scores, *n* = 102.

	Unstandardized Beta Coefficients	95.0% CI for Beta Coefficient		*p*-Value
		Lower Bound	Upper Bound	
(Constant)	22.413	10.761	34.065	<0.001
Setting = Nursing Home	−0.442	−2.821	1.937	0.713
Age (years)	−0.025	−0.144	0.094	0.679
Sex = Male	3.785	0.934	6.637	0.010
Marital state = widow/divorced	3.081	0.170	5.992	0.038
Physically aggressive behavior	6.302	4.072	8.532	<0.001
Physically non-aggressive behavior	−0.085	−2.564	2.393	0.946
Verbally agitated Behavior	2.662	0.450	4.874	0.019
Montreal Cognitive Assessment (MoCA)	−0.056	−0.355	0.243	0.712
Social Convoy (SCQ)	0.134	−1.015	1.282	0.818

DV = Negative affect score. Model adjusted R-squared = 0.297.

**Table 3:** Multivariate linear regression analysis of the dementia patients’ positive affect score, *n* = 102.

	Unstandardized Beta Coefficients	95.0% CI for Beta Coefficient		*p*-Value
		Lower Bound	Upper Bound	
(Constant)	26.393	11.924	40.862	<0.001
Settings = Nursing Homes	−5.784	−10.000	−1.568	0.008
Age (years)	−0.097	−0.243	0.048	0.187
Sex = Male	3.543	0.642	6.445	0.017
Income (JOD)	−0.025	−0.040	−0.011	0.001
Has a family with a medical/healthcare background	5.950	0.672	11.228	0.028
Educational level = diploma or higher education	5.372	0.434	10.310	0.033
Physically aggressive behavior	1.480	−1.396	4.356	0.309
Physically non-aggressive behavior	−0.735	−3.919	2.450	0.648
Verbally agitated behavior	−2.529	−5.354	0.297	0.079
Montreal Assessment (MoCA) scale total score	0.285	−0.096	0.666	0.141
Social Convoy (SCQ) overall mean score	1.276	−0.160	2.713	0.081

DV = Positive Affect score. Model adjusted R-squared = 0.131.

## Data Availability

The data that support the findings of this study are available on request from the corresponding author, [M.R.]. The data are not publicly available due to their containing information that could compromise the privacy of research participants.

## References

[1] Arvanitakis Z., Shah R.C., Bennett D.J.J. (2019). Diagnosis and management of dementia. JAMA.

[2] World Health Organization (2021). Global Status Report on the Public Health Response to Dementia.

[3] Cao Q., Tan C.C., Xu W., Hu H., Cao X.P., Dong Q., Tan L., Yu J.T. (2020). The prevalence of dementia: A systematic review and meta-analysis. J. Alzheimers Dis..

[4] Gotovac K., Perković M.N., Pivac N., Borovečki F.J.P.I.N.-P., Psychiatry B. (2016). Biomarkers of aggression in dementia. Prog. Neuropsychopharmacol. Biol. Psychiatry.

[5] Khan S.S., Ye B., Taati B., Mihailidis A. (2018). Detecting agitation and aggression in people with dementia using sensors—A systematic review. Alzheimers Dement..

[6] Kurisu K., Terada S., Oshima E., Horiuchi M., Imai N., Yabe M., Yokota O., Ishihara T., Yamada N. (2016). Comparison of QOL between patients with different degenerative dementias, focusing especially on positive and negative affect. Int. Psychogeriatr..

[7] Liu H., Zhang Z., Choi S.-W., Langa K.M. (2020). Marital status and dementia: Evidence from the Health and Retirement Study. J. Gerontol. B Psychol. Sci. Soc. Sci..

[8] Mejía S.T., Hooker K.J.P. (2015). Emotional well-being and interactions with older adults’ close social partners: Daily variation in social context matters. Psychol. Aging.

[9] Fingerman K.L., Kim Y.K., Ng Y.T., Zhang S., Huo M., Birditt K.S.J.T.G. (2022). Television viewing, physical activity, and loneliness in late life. Gerontologist.

[10] Lee K.H., Boltz M., Lee H., Algase D.L. (2017). Does social interaction matter psychological well-being in persons with dementia?. Am. J. Alzheimers Dis. Other Demen..

[11] Al Ghassani A., Rababa M.J. (2022). Size of Core Network: Why Less May Be More for Older Adults with Dementia. Ageing Int..

[12] Al Ghassani A., Rababa M.J.D., Disorders G.C. (2021). The Relationship between Size of Core Network and Frequency of Contacts with Agitation and Positive Affect in Older Adults with Dementia. Dement. Geriatr. Cogn. Disord..

[13] Jao Y.L., Loken E., MacAndrew M., Van Haitsma K., Kolanowski A. (2018). Association between social interaction and affect in nursing home residents with dementia. Aging Ment. Health.

[14] Allen A.P., Curran E.A., Duggan Á., Cryan J.F., Chorcoráin A.N., Dinan T.G., Molloy D.W., Kearney P.M., Clarke G. (2017). A systematic review of the psychobiological burden of informal caregiving for patients with dementia: Focus on cognitive and biological markers of chronic stress. Neurosci. Biobehav. Rev..

[15] Resnick B., Van Haitsma K., Kolanowski A., Galik E., Boltz M., Zhu S., Ellis J., Behrens L., Eshraghi K. (2021). Implementation of the Evidence Integration Triangle for behavioral and psychological symptoms of dementia (EIT-4-BPSD) in care communities. Nurs. Outlook.

[16] Hussein S., Ismail M. (2017). Ageing and elderly care in the Arab region: Policy challenges and opportunities. Ageing Int..

[17] Nasreddine Z.S., Phillips N.A., Bédirian V., Charbonneau S., Whitehead V., Collin I., Cummings J., Chertkow H. (2005). The Montreal Cognitive Assessment, MoCA: A brief screening tool for mild cognitive impairment. J. Am. Geriatr. Soc..

[18] Cohen-Mansfield J., Werner P., Marx M.S. (1989). An observational study of agitation in agitated nursing home residents. Int. Psychogeriatr.

[19] Watson D., Clark L.A., Tellegen A. (1988). Development and validation of brief measures of positive and negative affect: The PANAS scales. J. Pers. Soc. Psychol..

[20] Crawford J.R., Henry J.D. (2004). The Positive and Negative Affect Schedule (PANAS): Construct validity, measurement properties and normative data in a large non-clinical sample. Br. J. Clin. Psychol..

[21] Antonucci T.C., Akiyama H. (1987). Social networks in adult life and a preliminary examination of the convoy model. J. Gerontol..

[22] Dunn T.W., Burlingame G.M., Walbridge M., Walbridge M., Crum M. (2005). Outcome assessment for children and adolescents: Psychometric validation of the youth outcome questionnaire 30.1 (Y-OQ^®^-30.1). Clin. Psychol. Psychother..

[23] Polit D.F., Beck C.T. (2010). Essentials of Nursing Research: Appraising Evidence for Nursing Practice.

[24] Gliem J.A., Gliem R.R. Calculating, interpreting, and reporting Cronbach’s alpha reliability coefficient for Likert-type scales. Proceedings of the 2003 Midwest Research to Practice Conference in Adult, Continuing, and Community Education, Presented at the Midwest Research-to-Practice Conference in Adult, Continuing, and Community Education.

[25] Sattler C., Toro P., Schönknecht P., Schröder J.J.P.R. (2012). Cognitive activity, education and socioeconomic status as preventive factors for mild cognitive impairment and Alzheimer’s disease. Psychiatry Res..

[26] Zunzunegui M.V., Alvarado B.E., Del Ser T., Otero A. (2003). Social networks, social integration, and social engagement determine cognitive decline in community-dwelling Spanish older adults. J. Gerontol. B Psychol. Sci. Soc. Sci..

[27] Hirosaki M., Ishimoto Y., Kasahara Y., Konno A., Kimura Y., Fukutomi E., Chen W., Nakatsuka M., Fujisawa M., Sakamoto R. (2013). Positive affect as a predictor of lower risk of functional decline in community-dwelling elderly in Japan. Geriatr. Gerontol. Int..

[28] Murata A., Mayumi T., Muramatsu K., Ohtani M., Matsuda S., Research E. (2015). Effect of dementia on outcomes of elderly patients with hemorrhagic peptic ulcer disease based on a national administrative database. Aging Clin. Exp. Res..

[29] Murata A., Mayumi T., Muramatsu K., Ohtani M., Matsuda S. (2009). Predictors of maintaining cognitive function in older adults: The Health ABC study. Neurology.

[30] Pillemer S., Davis J., Tremont G.J.A., Health M. (2018). Gender effects on components of burden and depression among dementia caregivers. Aging Ment. Health.

[31] Masumoto K., Taishi N., Shiozaki M.J.G., Medicine G. (2016). Age and gender differences in relationships among emotion regulation, mood, and mental health. Gerontol. Geriatr. Med..

[32] Barnes D.E., Yaffe K. (2011). The projected effect of risk factor reduction on Alzheimer’s disease prevalence. Lancet Neurol..

[33] Nikmat A.W., Al-Mashoor S.H., Hashim N. (2015). Quality of life in people with cognitive impairment: Nursing homes versus home care. Int. Psychogeriatr..

[34] Siu O.-L., Phillips D., Development H. (2002). A study of family support, friendship, and psychological well-being among older women in Hong Kong. Int. J. Aging Hum. Dev..

[35] Bae J.B., Kim Y.J., Han J.W., Kim T.H., Park J.H., Lee S.B., Lee J.J., Jeong H.G., Kim J.L., Jhoo J.H. (2015). Incidence of and risk factors for Alzheimer’s disease and mild cognitive impairment in Korean elderly. Dement. Geriatr. Cogn. Disord..

[36] Sommerlad A., Ruegger J., Singh-Manoux A., Lewis G., Livingston G. (2018). Marriage and risk of dementia: Systematic review and meta-analysis of observational studies. J. Neurol. Neurosurg. Psychiatry.

[37] Cohen-Mansfield J., Dakheel-Ali M., Jensen B., Marx M.S., Thein K. (2012). An analysis of the relationships among engagement, agitated behavior, and affect in nursing home residents with dementia. Int. Psychogeriatr..

[38] Hwang T.J., Masterman D.L., Ortiz F., Fairbanks L.A., Cummings J.L. (2004). Mild cognitive impairment is associated with characteristic neuropsychiatric symptoms. Alzheimer Dis. Assoc. Disord..

[39] Kunik M.E., Snow A.L., Davila J.A., McNeese T., Steele A.B., Balasubramanyam V., Doody R., Schulz P.E., Kalavar J.S., Walder A. (2010). Consequences of aggressive behavior in patients with dementia. J. Neuropsychiatry Clin. Neurosci..

[40] Kales H.C., Gitlin L.N., Lyketsos C.G. (2015). Assessment and management of behavioral and psychological symptoms of dementia. BMJ.

[41] Benninghoff J., Perneczky R. (2022). Anti-Dementia Medications and Anti-Alzheimer’s Disease Drugs: Side Effects, Contraindications, and Interactions. NeuroPsychopharmacotherapy.

[42] Pulsford D., Duxbury J., Nursing M.H. (2006). Aggressive behaviour by people with dementia in residential care settings: A review. J. Psychiatr. Ment. Health Nurs..

